# Astrocytes have the capacity to act as antigen-presenting cells in the Parkinson’s disease brain

**DOI:** 10.1186/s12974-020-01776-7

**Published:** 2020-04-16

**Authors:** Jinar Rostami, Grammatiki Fotaki, Julien Sirois, Ropafadzo Mzezewa, Joakim Bergström, Magnus Essand, Luke Healy, Anna Erlandsson

**Affiliations:** 1 Molecular Geriatrics, Department of Public Health and Caring Sciences/Molecular Geriatrics, Rudbeck Laboratory, Uppsala University, SE-751 85 Uppsala, Sweden; 2 Department of Immunology, Genetics and Pathology, Uppsala University, SE-751 85 Uppsala, Sweden; 3 Neuroimmunology Unit, department of Neurology and Neurosurgery, Montreal Neurological Institute, McGill University, Montreal, 3801 Canada

**Keywords:** Astrocytes, T-cell infiltration, MHC-II, Antigen presentation, Alpha-synuclein, Parkinson’s disease, Tunneling nanotubes

## Abstract

**Background:**

Many lines of evidence suggest that accumulation of aggregated alpha-synuclein (αSYN) in the Parkinson’s disease (PD) brain causes infiltration of T cells. However, in which ways the stationary brain cells interact with the T cells remain elusive. Here, we identify astrocytes as potential antigen-presenting cells capable of activating T cells in the PD brain. Astrocytes are a major component of the nervous system, and accumulating data indicate that astrocytes can play a central role during PD progression.

**Methods:**

To investigate the role of astrocytes in antigen presentation and T-cell activation in the PD brain, we analyzed *post mortem* brain tissue from PD patients and controls. Moreover, we studied the capacity of cultured human astrocytes and adult human microglia to act as professional antigen-presenting cells following exposure to preformed αSYN fibrils.

**Results:**

Our analysis of post mortem brain tissue demonstrated that PD patients express high levels of MHC-II, which correlated with the load of pathological, phosphorylated αSYN. Interestingly, a very high proportion of the MHC-II co-localized with astrocytic markers. Importantly, we found both perivascular and infiltrated CD4^+^ T cells to be surrounded by MHC-II expressing astrocytes, confirming an astrocyte T cell cross-talk in the PD brain. Moreover, we showed that αSYN accumulation in cultured human astrocytes triggered surface expression of co-stimulatory molecules critical for T-cell activation, while cultured human microglia displayed very poor antigen presentation capacity. Notably, intercellular transfer of αSYN/MHC-II deposits occurred between astrocytes via tunneling nanotubes, indicating spreading of inflammation in addition to toxic protein aggregates.

**Conclusions:**

In conclusion, our data from histology and cell culture studies suggest an important role for astrocytes in antigen presentation and T-cell activation in the PD brain, highlighting astrocytes as a promising therapeutic target in the context of chronic inflammation.

## Background

Parkinson’s disease (PD) is a progressive neurodegenerative disorder with complex pathophysiology that develops over decades. The main characteristics of PD are intracellular deposits of aggregated αSYN and chronic neuroinflammation [1, 2]. Nonetheless, the interplay between these two processes and their respective impact on disease progression remain elusive. A recent study showed that CD4^+^ T helper cells derived from PD patients recognize αSYN fibrils as an antigen [3]. In accordance, the presence of infiltrating T cells has been confirmed in animal models of PD as well as in post mortem brain tissues of PD patients [4–6]. However, which cell types that act as antigen-presenting cells (APCs) in the PD brain and activate the infiltrating CD4^+^ T cells remain unclear.

Astrocytes, the most numerous glial cell type in the central nervous system, are crucial for maintaining brain homeostasis, but are also highly involved in neuroinflammation [7]. In the PD brain, astrocytes are converted to a reactive, inflammatory state in which they phagocytose aggregated proteins and cell debris as well as secrete various cytokines and chemokines [8, 9]. Moreover, αSYN deposits appear frequently in astrocytes at all disease stages [10–14]. We have previously demonstrated that αSYN accumulation in cultured human astrocytes result in severe cellular stress, indicated by lysosomal, mitochondrial, and endoplasmic reticulum deficiencies. The stressed astrocytes respond by sending out tunneling nanotubes, enabling intercellular transfer of αSYN inclusions to nearby cells, indicating astrocytes as crucial players in the progression of PD pathology [15, 16].

All nucleated cells in the body express major histocompatibility class I (MHC-I) for presentation of antigenic peptides to CD8^+^ T cells, while so called antigen-presenting cells (APCs) also express MHC class II (MHC-II), enabling them to present antigenic peptides to CD4^+^ helper T cells. Dendritic cells (DCs), B cells, and macrophages are considered classical or professional APCs as they also express the co-stimulatory molecules, including CD80, CD86, and CD40, that are required for activation of CD8^+^ T cells and CD4^+^ T cells [17]. The cell surface expression of the co-stimulatory factors by the professional APCs can for example be induced by the cytokine interferon-gamma (IFNγ). APCs degrade ingested antigens via the endo-lysosomal pathway and digest the antigen to smaller fragments that are presented on MHC-II. It is believed that DCs are required for activation of naive T cells and a cytolytic T-cell mediated immune response as they can also cross-present exogenously ingested peptides on their MHC-I for activation of CD8^+^ T cells. Another feature of professional APCs is their ability to cross-talk and transfer MHC-II molecules between each other via cell-to-cell transfer [18].

For decades, there has been a debate whether APCs are present in the brain. Microglial cells arise from the yolk sac and invade the brain during embryonic development. They have been considered to be the resident CNS macrophages and consequently presumed to be the most likely brain APCs. However, several studies indicate that microglia are in fact poor antigen presenters [19–21]. Our previous observations that human astrocytes accumulate and transfer aggregated αSYN raised the question whether infiltrating T cells interact with the αSYN containing astrocytes and if the astrocytes can act as APCs in the PD brain. Hence, the aim with the present study was to investigate the role of αSYN accumulating astrocytes in the chronic neuroinflammation in PD, in regards to the adaptive immunity.

## Materials and methods

### Immunohistochemistry

#### Deparaffinization

Brain sections from mesencephalon and striatum of 10 PD patients and 10 controls (Table 1) were used (kindly provided from Uppsala Biobank by Professor Irina Alafuzoff, ethical number 2012/494). The thickness of the analyzed sections was 5 μm. To insure that exactly the same region was analyzed from all patients and controls, a cutting mold was used during the sectioning procedure. Prior to immunostaining, the sections were deparaffinized using xylene 2× for 10 min, rehydrated using 99.5% ethanol 2× for 10 min, 95% ethanol for 10 min, and 70% ethanol for 10 min and washed in PBS 3 × 5 min.

**Table 1:** Sex, age, and post mortem fixation time of the 10 PD patients and the 10 controls analyzed in this study

PD			Control		
Sex	Age	Post mortem fixation time (weeks)	Sex	Age	Post mortem fixation time (weeks)
Male	83	6	Male	72	4
Male	84	4	Male	67	4
Male	74	4	Male	72	5
Male	77	3	Male	71	3
Male	89	3	Male	68	5
Male	74	3	Male	61	5
Male	81	4	Male	75	5
Male	79	5	Female	77	5
Female	80	5	Female	61	6
Female	78	4	Female	75	6

#### Antigen retrieval and permeabilization

Antigen retrieval was performed by boiling the samples in citric acid at pH 6 for 2 min in 550 W. The samples were left to cool down in room temperature (RT) for 20–30 min before permeabilization with 0.4% triton for 20 min. Endogenous peroxidase was blocked with 1% H_2_O_2_ (Thermo Fischer) for 30 min and blocked with TNB blocking buffer (0.1 M Tris-HCl and 0.15 M NaCl and 0.5% blocking reagent FP1020, Perkin Elmer) for 30 min.

#### Primary antibody incubation

Primary antibodies were prepared in PBS and incubated with the sections at 4 °C overnight. The primary antibodies used were HLADR/DP/DQ (DAKO M0775, 1:200), glial fibrillary acidic protein (GFAP, Abcam ab4674, 1:500), ionized calcium-binding adaptor molecule 1 (Iba1, Abcam ab178846, 1:400), glutamate aspartate transporter (GLAST, Novus biologicals NB100-1869, 1:400) phosphorylated ser 129 αSYN (p-αSYN) (Abcam ab 51253, 1:500), Clone 42 (BD Biosciences, 610787, 1:200), and CD4 (Abcam ab133616, 1:100). The following day, the sections were washed in PBS 3 × 5 min.

#### Secondary antibody incubation

For the tyramide signal amplification kit (TSA), HRP-conjugated secondary antibodies were used. A maximum of three primary antibodies were used for one staining, and the sections were incubated with one secondary antibody at the time for 1 h at RT. Thereafter, the sections were washed in PBS 3 × 5 min. For development, TSA reagent coupled with either Cy3.5 FITC or Cy5 (Perkin Elmer, 1:100 in amplification buffer) was added to the tissue sections for 15 min at RT in the dark. Before incubation with the next secondary antibody, the HRP activation from the previous staining was abolished using 0.4% sodium azide for 10 min, followed by washes and incubation with 1% H_2_O_2_ for 15 min. The sections were blocked with TNB blocking buffer for 30 min. After incubation and development of all secondary antibodies, the sections were dipped in 70% ethanol five times, incubated in 0.3% Sudan black for 20 min, dipped in 70% ethanol twelve times, washed in PBS, and mounted using EverBrite Hardset mounting medium (Bionordika).

### Production of αSYN fibrils

#### Generation

Endotoxin-free recombinant human αSYN (Anaspec A5555-1000) was resolved in sterile PBS to a concentration of 5 mg/ml and incubated on a shaker (IKA MS3 Basic, 1000 rpm) at 37 °C for 7 days to form αSYN preformed fibrils (αSYN-F). Thereafter, the αSYN-F were diluted to a working concentration of 2 mg/ml (140 μM) and left in − 70 °C until use.

#### Characterization

The αSYN-F were characterized using centrifugation, Thioflavin T (ThT) assay, and electron microscopy (EM). A short centrifugation at ×16,000 *g* for 5 min was performed to ensure pellet formation of insoluble, large fibrils. ThT solution (100 μM) was added to recombinant monomers (αSYN-M) (diluted 1:100 from 2 mg/ml in PBS), αSYN-F (diluted 1:100 from 2 mg/ml in PBS), and read instantly at a wave length of 420 nm. For electron microscopy, αSYN-M, αSYN-F, and sonicated αSYN-F (all diluted 1:5 in PBS) were dropped onto carbon coated 300-mesh copper grids, negatively stained with 1% uranyl acetate for 5 min and air dried. The samples were analyzed using a Hitachi H-7100 transmission electron microscope.

#### Cy3 labeling

αSYN-F and αSYN-M were labeled with Cy3^AM^ antibody labeling kit (GE Healthcare, PA33000) for 1 h, according to the manufacture’s protocol. To remove the unbound Cy3, the αSYN-F were centrifuged at ×23,000*g* for 30 min, the supernatant was removed, and the pellet was resolved in sterile PBS. The washing procedure was repeated three times.

#### Sonication

αSYN-F were diluted 1:2 in sterile PBS and sonicated in 20% amplitude, 1 s off and 1 s on for 30 s using a Sonics Vibra Cell sonicator, immediately prior to the experiment.

### Culture of human astrocytes

Human embryonic stem cell (ESC)-derived astrocytes [22] were expanded in Advanced DMEM/F12 (Thermo Fischer 12634-010) supplemented with 20 ng/ml Human Ciliary Neurotrophic Factor (CNTF) (Thermo Fischer), 1% penicillin streptavidin (Thermo Fischer 15140-122), 1% B27 supplement (Thermo Fischer 17504-044), 1% non-essential amino acids (Merc Millipore TMS001-C), and 1% l-glutamine (Thermo Fischer 25030-024). Cells were passaged using Trypsin-EDTA (Life technologies). Two weeks prior to the experiments, the media was changed to Neurobasal medium supplemented with 20 ng/ml CNTF, 1% penicillin streptavidin, 1% non-essential amino acids, 1% l-glutamine (Thermo Fischer 25030-024), and N2 supplements (Thermo Fischer 17502048). For experiments, the astrocytes were seeded at a concentration of 4500 cells/cm^2^, resulting in a 40–50% confluence. Cells from 90 DIV to 120 DIV were used in the study.

### Isolation and culture of adult human microglia

Human adult microglia were isolated as described previously [23] (ethical number ANTJ 1988/3). Shortly, human brain tissue was obtained from the temporal lobe of three epilepsy patients undergoing surgery. A separate microglia culture was derived from each donor. The tissue was washed in PBS several times, and blood clots were removed using a Pasteur pipette. The tissue was then incubated with 0.5% trypsin and 25 μg/ml of DNase in PBS on shake for 30 min at 37 °C. The trypsin was inactivated using 10% fetal calf serum (FCS), and the tissue was dissociated using a mashing filter prior to centrifugation at 1200 rpm for 10 min. Thereafter, the cells and debris were resuspended in PBS and added to Percoll before centrifugation at ×31,000*g* for 30 min with no breaks at 4 °C. The top layer containing myelin was removed, and the middle layer consisting of microglia and oligodendrocytes was resuspended in PBS and centrifuged at 1200 rpm for 10 min. The cells were counted and cultured at a density of 2 × 10^6^ cells/ml in a T12.5 flask in MEM medium supplemented with 5% FCS, 0.1% glucose, 1% penicillin-streptomycin, and 1% glutamine. Following day, the floating cells (which are oligodendrocytes) were removed. The cells were passaged using 0.05% trypsin and 2 mM EDTA and cultured at a density of 50,000 cells/cm^2^ for experiment.

### Astrocytes and microglia exposure to αSYN-F, αSYN-M and IFNγ

Astrocyte and microglia cultures were exposed to 0.5 μM sonicated αSYN-F (Cy3 labeled or unlabeled), IFNγ (Thermo Fischer 100 U/ml for astrocytes and 25 U/ml for microglia), 0.5 μM αSYN-F + IFNγ, and 0.5 μM αSYN-M or were left untreated. After 7 days of exposure the cells were fixed or detached for further analyses.

### Immunocytochemistry

Cells were fixed in 4% paraformaldehyde (PFA) in PBS and washed with PBS. Blocking and permeabilization were performed with 5% normal goat serum (NGS) and 0.1% triton in PBS for 30 min in RT. Primary antibodies were diluted in 0.5% NGS and 0.1% triton in PBS and added to the cells for 2 h at RT. Thereafter, cells were washed 3 times with PBS prior to incubation with secondary antibodies or dyes for 1 h at RT. After additional washes, the cells were mounted, using Ever Brite Hardset Mounting medium with or without DAPI (BioNordika). The cells were analyzed using the fluorescence microscope Observer and Z1 Zeiss and confocal images were taken using Zeiss LSM700. The primary antibodies used were CD68 (DAKO, m0841, Clone KP1, 1:200), MHC-II (DAKO M0775, 1:200), MHC-I (Abcam EMR8-5 1:100), PDL1-1 (Novus biologicals NBP-176769 1:200), LAMP-1 (Abcam ab24170 1:200), nestin (Millipore ABD69 1:200), vimentin (Millipore AB5733 1:200), GLAST (Novus biologicals NB100-1869 1:400), and ALDH1L1 (Novus biologicals NBP2-24143 1:400). The secondary antibodies and dyes used were Alexaflour 488 goat anti rabbit/mouse (Thermo Fischer, 1:200) and Alexaflour 555 goat anti rabbit/mouse (Thermo Fischer, 1:200). To stain the plasma membrane, cells were incubated with wheat germ agglutinin and Alexa Fluor® 350 Conjugate (Life technologies, 1:200) for 10 min.

### Flow cytometry on astrocytes

The cells were detached using Accutase (Thermo Fischer) and centrifuged in ×300*g* for 5 min. The pelleted cells were resuspended in staining buffer containing 2% FBS (BD Biosciences) and Versen (Thermo Fishcer) (1:1), before incubation with antibodies for 20 min in the dark. The following antibodies from BD Biosciences were used according to manufacturer’s instructions: APC mouse anti-human CD40 (555591), BV510 mouse anti-human CD80 (563084), PE mouse anti-human CD86 (555665), FITC mouse anti-human HLADR/DP/DQ (555558), PE-Cy7 mouse anti-human HLA-A/B/C (561349), and BV421 mouse anti-human PDL-1 (563738). The cells were washed and resuspended in Versen, and the fluorescence signal was examined with the machine flow cytometry Canto II (BD Biosciences). The flow cytometry data was analyzed using the FlowJo software (version 7.6.5; Tree Star, Ashland, OR) software.

### Flow cytometry on microglia

Microglia cultures were detached using Accutase (Thermo Fischer) and centrifuged at ×300*g* for 5 min. The cells were incubated in blocking buffer (PBS, 10% normal human serum and normal mouse IgG (3 mg/ml)) for 30 min and centrifuged at ×300*g* for 5 min, before incubation with the antibodies for 20 min in the dark. The following antibodies from BD Biosciences were used according to the manufacturer’s instructions: APC mouse anti-human CD86 (BD Biosciences; 555660), FITC mouse anti-human HLA-DR/DP/DQ (BD Biosciences; 555558), BV786 mouse anti-human CD80 (BD Biosciences; 564159), PerCp mouse anti-human CD40 (Biolegend; 334316), and BV650 mouse anti-human HLA-A/B/C (BD Biosciences; 740407). The cells were washed and resuspended in 2 mM EDTA in PBS, and the fluorescence signal was examined and analyzed using the FACSAria Fusion (BD Biosciences). The data was analyzed using the FlowJo software (version 7.6.5; Tree Star, Ashland, OR) software.

### Statistical analyses

#### Immunohistochemistry

For the MHC-II/GFAP/Iba1 immunostainings, mesencephalon and striatum sections from 10 PD patients and 10 controls were analyzed (Table 1). For the MHC-II/GLAST immunostainings, mesencephalon sections from 10 PD patients and 10 controls were analyzed. Forty images were captured from each sample and region.

##### MHC-II-integrated density measurement, correlation between mesencephalon and striatum and correlation with age

The raw integrated density (IntDen) (area × mean intensity) of the MHC-II signal was measured using ImageJ. The MHC-II expression between patient and control individuals was analyzed using Mann-Whitney *t* test in Graphpad. To analyze whether there was a correlation between the expression of MHC-II in mesencephalon versus striatum, linear regression was performed on the average IntDen of all the 40 images from each individual. To analyze whether there was a correlation between the expression of MHC-II and the age of the individuals in the PD group and in the control group, linear regression was performed on the average IntDen of the MHC II staining versus age.

##### Measurement of the GLAST^+^/ MHC-II^+^, GFAP^+^/ MHC-II^+^, and Iba1^+^/MHC-II^+^ area

The MHC-II^+^ and GLAST^+^ areas, the MHC-II^+^ and GFAP^+^ areas, and the MHC-II^+^, and Iba1^+^ and total MHC-II^+^ areas were measured in all images. Based on co-localization of MHC-II and GLAST, GFAP and Iba1 positive staining respectively, the percentage of GLAST^+^/MHC-II^+^, GFAP^+^/ MHC-II^+^, and Iba1^+^/MHC-II^+^ of the total MHC-II^+^ area was calculated. The comparison between the patient and control group was performed using Student *t* test. 

##### Correlation between MHC-II staining and p-ser 129 αSYN staining in mesencephalon

Mesencephalon sections were stained for p-ser 129 αSYN (p-αSYN), and forty images were taken from each patient. Using Image J, IntDen for p-αSYN was measured. Non-linear regression with exponential model was performed on the MHC-II and the p-αSYN IntDen.

#### Cell culture experiments

All data presented are based on analyses of three independent cell culture experiments.

#### Immunocytochemistry

The human astrocytes were stained with specific antibodies for LAMP-1, MHC-II, MHC-I, and PDL-1. From each experiment, 12 images were captured for each marker.

#### Flow cytometry

The flow cytometry data was analyzed using ANOVA with Bonferroni correction in GraphPad Prism.

## Results

### The majority of the MHC-II in the PD brain overlaps with astrocytic markers

**Fig. 1:**
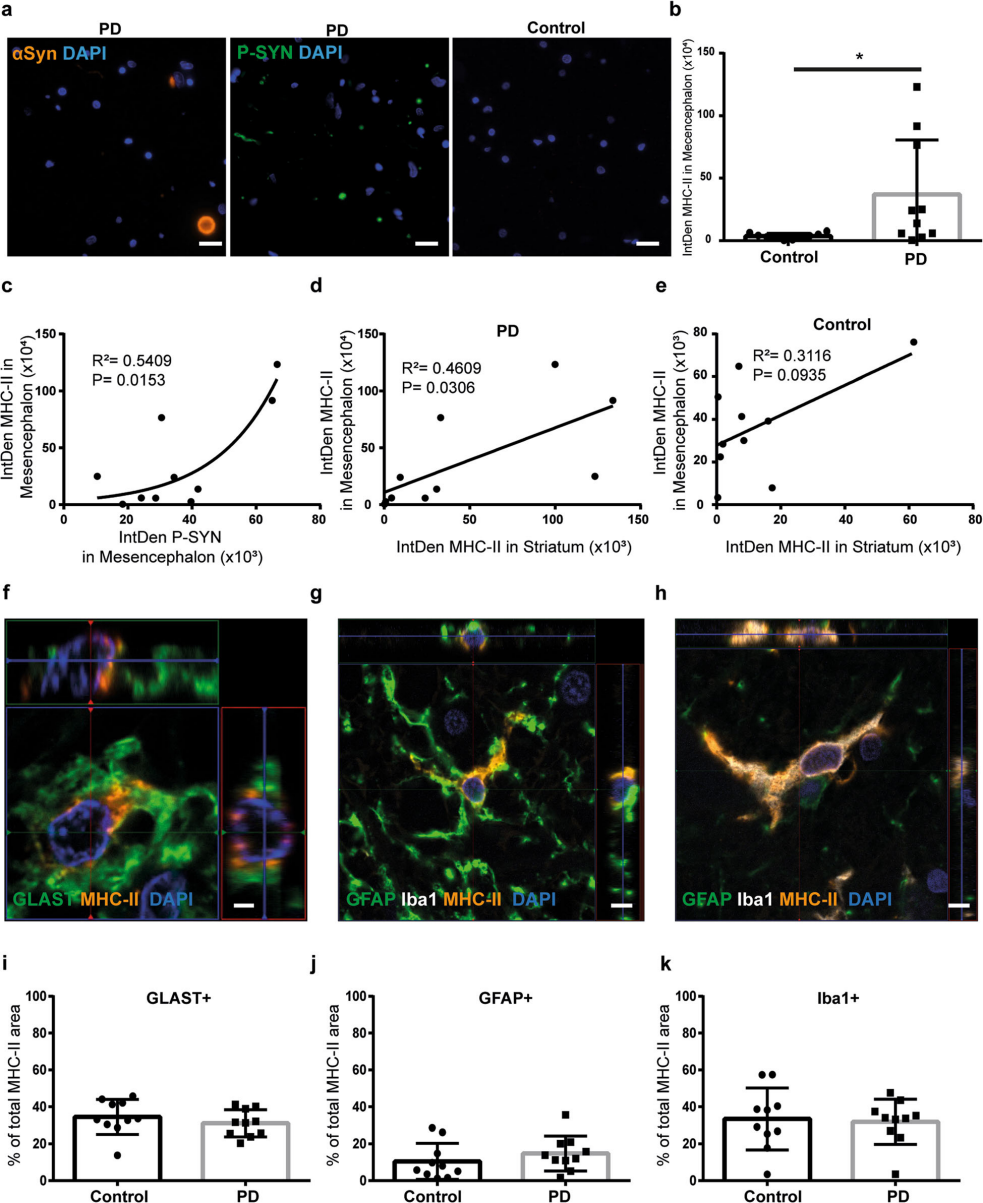
Human astrocytes express MHC-II in brain tissue from PD patients and healthy controls**.** The αSYN pathology was confirmed in the PD cases by immunostaining of αSYN and phosphorylated αSYN (p-αSYN) (**a**). The PD brains express higher levels of MHC-II compared to control brains (**b**). MHC-II levels correlate with the expression of p-αSYN in PD brains (**c**). There is a correlation between the MHC-II levels in the mesencephalon and striatum in the PD brains, but not in the control brains (**d**, **e**). Astrocytes labeled with GLAST (**f**) or GFAP (**g**) are expressing high levels of MHC-II, as well as Iba1-labeled microglia (**h**). Quantifications of the immunostainings revealed that 31% of the MHC-II area overlapped with the astrocyte marker GLAST (**i**), 15% overlapped with the reactive astrocyte marker GFAP (**j**), and 32 % overlapped with the microglia marker Iba1 in the PD brain (**k**). A similar overlap between MHC-II and GLAST, GFAP, and Iba1 (35%, 10%, and 33%, respectively) was revealed from the analysis of the control brains (**j**–**k**). Scale bars: **a** = 20 μm; **f** = 2 μm; **g**, **h** = 5 μm

We first sought to investigate if there was a correlation between PD pathology and the expression of MHC-II. The presence of Lewy body pathology in PD cases and the absence of pathology in the control cases were confirmed by staining brain sections with αSYN and p-αSYN antibodies (Fig. 1a). In order to investigate if the levels of MHC-II are different between PD brains and control brains, we performed immunostainings for MHC-II and p-αSYN. Quantifications of MHC-II IntDen (area × mean intensity) in mesencephalon demonstrated a significant increase of MHC-II expression in PD patients compared to healthy controls (Fig. 1b). Interestingly, we could observe a clear correlation between MHC-II expression and the presence of p-αSYN in the PD brains (Fig. 1c). Subsequently, we performed immunostainings for MHC-II in striatum to investigate if there was a correlation between MHC-II expression in different regions of the nigrostriatal pathway, which is heavily affected by αSYN pathology in PD. Indeed, we found a correlation of MHC-II expression between mesencephalon and striatum in the PD brains, but not in the control brains (Fig. 1d, e). To clarify whether the age of the patients had any impact on the MHC-II expression, we performed linear regression on the MHC-II and the age in the PD group and in the control group. Our analysis demonstrates that there is no correlation between age and the expression of MHC-II in the material included in this study (61–84 years of age) (Additional file 1 a and b).

Moreover, the immunohistochemical staining showed that MHC-II co-localized with the astrocytic markers GLAST (Fig. 1f) and GFAP (Fig. 1g), as well as with the microglial marker Iba1 (Fig. 1h) in both PD and control brain sections, demonstrating that both microglia and astrocytes express MHC-II. To analyze the frequency of MHC-II expression in the various cell types, the respective overlaps of MHC-II with GLAST, GFAP, and Iba1 were quantified. In both PD and control brains, one third (35 and 31 %, respectively) of the MHC-II area overlapped with GLAST^+^ astrocytes (Fig. 1i). There was no significant difference in the total GLAST^+^ area between controls and patients, confirming GLAST expression by both nonreactive and reactive astrocytes (Additional file 2a). Moreover, 15% of the MHC-II area overlapped with reactive GFAP^+^ astrocytes in PD brains and 10% in control brains (Fig. 1j). Importantly, GFAP stains the cytoskeleton of the astrocyte, which only constitutes 15–20% of the cell [24]. Hence, the whole astrocyte is much bigger than the GFAP staining, and the overlap with MHC-II and GFAP will only reflect a proportion of the MHC-II expressing reactive astrocytes. In contrast to GLAST, there was an increase in GFAP^+^ area in PD patients compared to controls (Additional file 2 a). Double staining of GLAST and GFAP (Additional file 2b) show that the two markers overlap very little, which was expected since they label different parts of the astrocyte. While GFAP is a cytoskeleton protein, GLAST is expressed in the plasma membrane of the cells. Moreover, the expression levels of GFAP and GLAST vary between different astrocytic subclasses. Hence, the combined result from the MHC-II/GLAST and MHC-II/GFAP staining show that almost 50% of the MHC-II overlapped with either of the astrocytic markers. Both in PD and control brains, approximately one third (33% and 32%, respectively) of the MHC-II area overlapped with Iba1 (Fig. 1k). Similar to GFAP, the total Iba1 area was increased in PD patients, compared to controls (Additional file 2a). The remaining MHC positive cells (approximately 20%) may reflect MHC-II^+^ infiltrating immune cells and/or GLAST-GFAP-MHC-II+ astrocytes. Hence, although the expression of MHC II is increased in the PD brain, the overlap with astrocytic markers and microglial markers is not significantly different. The most likely explanation is that the same cells express MHCII in the healthy brain and in the PD brain, but that their expression level is higher in the PD brain.

### Exposure to αSYN-F does not result in surface expression of co-stimulatory molecules in human microglia

**Fig. 2:**
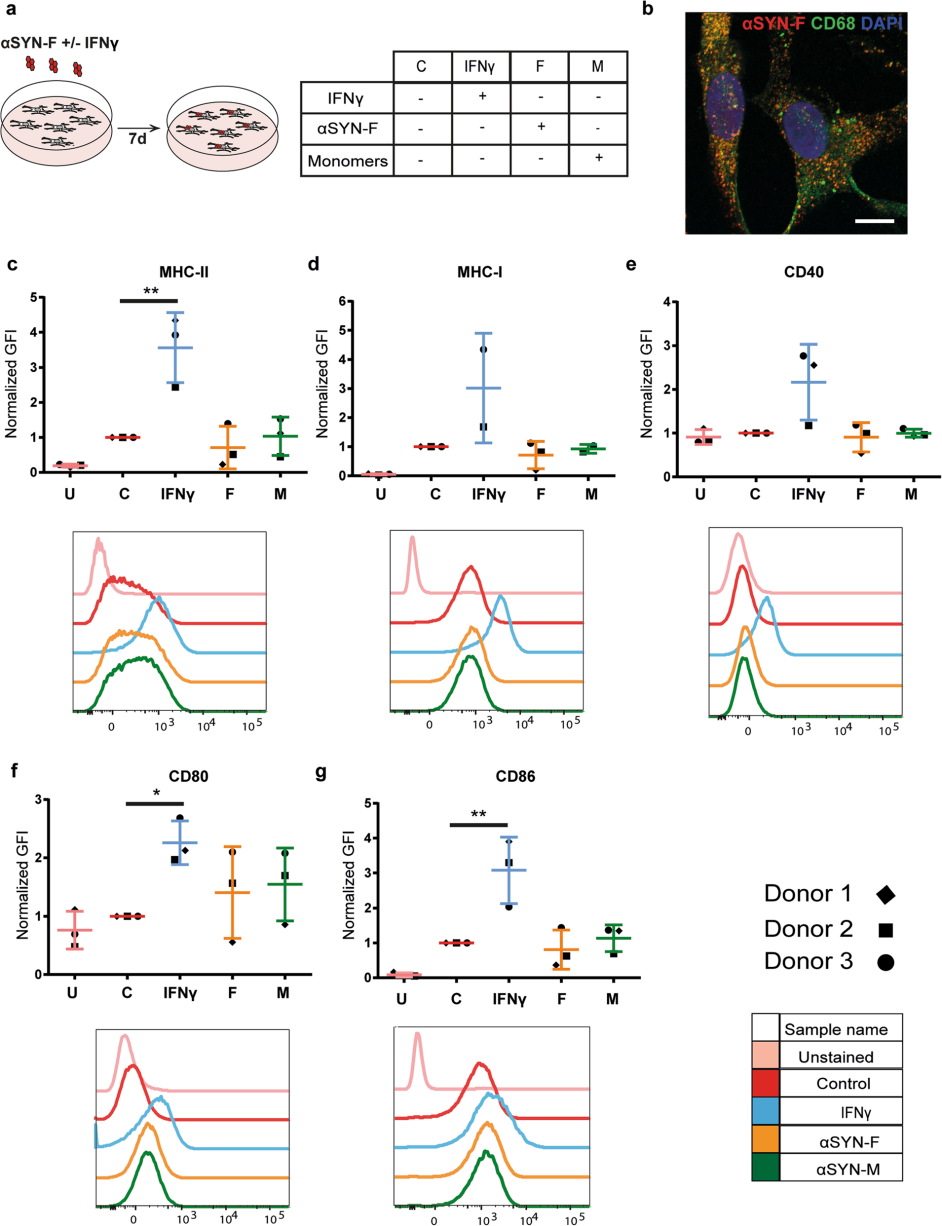
Human microglia do not express markers required for activation of CD4^+^ T cells, following αSYN-F treatment. Schematic illustration of study setup and the different treatment groups (**a**). Human microglia accumulate αSYN that partially co-localize with the lysosomal marker CD68 (**b**). Flow cytometry analysis revealed that microglia cells upregulate MHC-II (**c**) and MHC-I (**d**) upon IFNγ treatment,which are involved in activation of T cells. However, when exposed to αSYN-F, the microglia did not express co-stimulatory markers CD40 (**e**), CD80 (**f**), and CD86 (**g**) that are all necessary for activating CD4^+^ T cells. U, unstained samples; GFI, geometric fluorescence intensity

Due to their myeloid origin, microglia cells have been addressed as the primary antigen-presenting cells in the brain. To investigate antigen presentation capacities of microglia in PD, adult human microglia were exposed to sonicated αSYN preformed fibrils (αSYN-F) (Fig. 2a). The β-sheet structure of the αSYN-F was assessed with ThT assay (Additional file 3a), and TEM was performed to compare the αSYN-F before and after the sonication (Additional file 3b-c). Immunostaining for the lysosomal marker CD68, a phagocytic marker specific for microglial cells, illustrated intracellular accumulation of αSYN 7 d after exposure (Fig. 2b). Flow cytometry analysis showed upregulation of surface expression of MHC-I and MHC-II and the co-stimulatory molecules CD80, CD86, and CD40 (Fig. 2c–g) upon treatment with IFNγ, verifying that the microglia were healthy and responded normally to surrounding inflammatory signals. However, exposure of the microglia to αSYN-F did not result in increased surface expression of MHC-I and MHC-II or any of the co-stimulatory molecules important for T-cell activation (Fig. 2c–g). These results indicate that microglia cells are not capable of activating T cells following exposure to αSYN-F.

### Human astrocytes express co-stimulatory molecules following exposure to αSYN-F

**Fig. 3:**
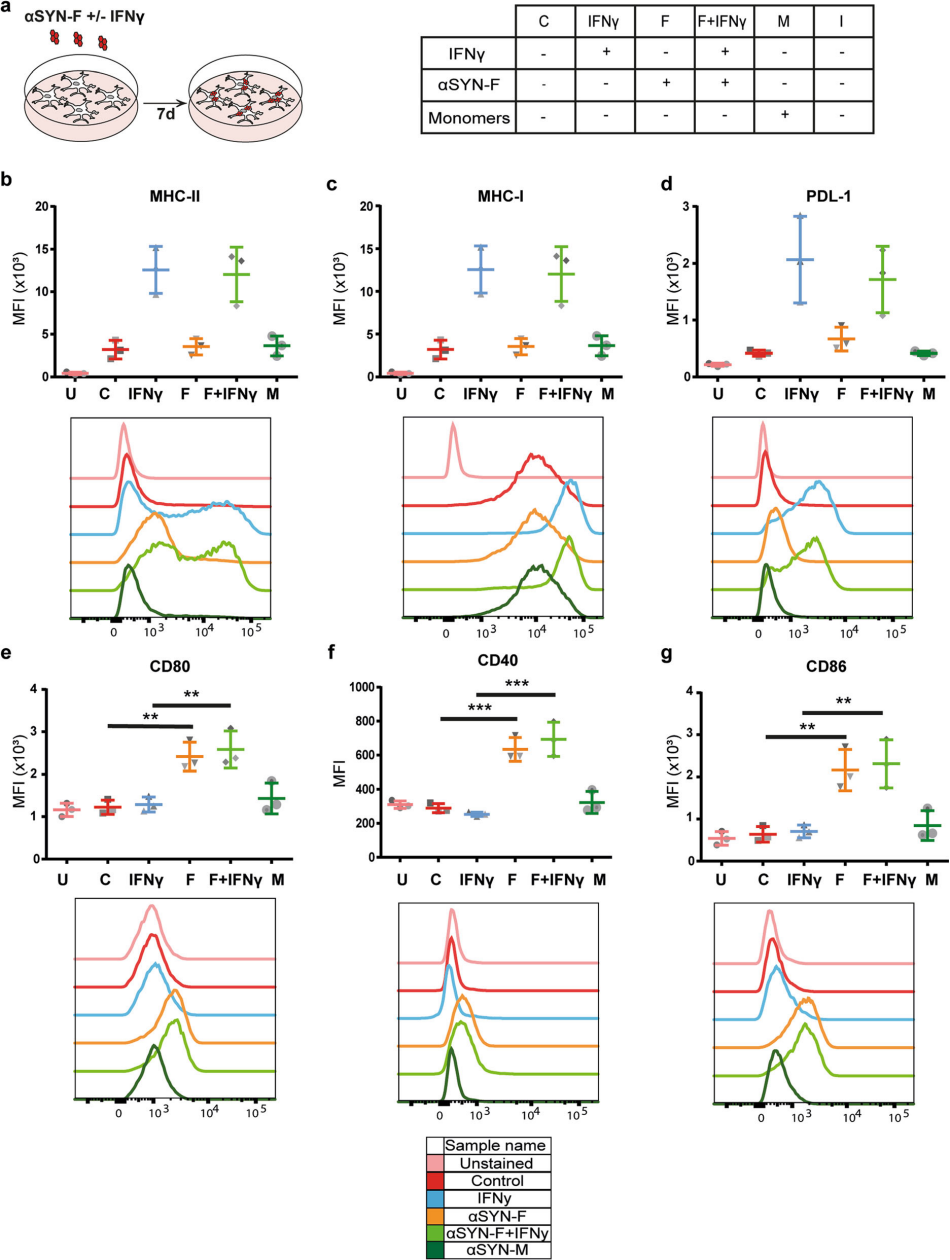
Human astrocytes express all markers required for activation of CD4^+^ T cells following αSYN-F treatment**.** Schematic illustration of study setup and the different treatment groups (**a**)**.** Flow cytometry analysis illustrated that astrocytes upregulate MHC-II (**b**), MHC-I (**c**), and PDL1 (**d**) upon IFNγ treatment ,which are involved in activation (MHC-II and MHC-I) and inhibition (PDL1) of T cells. Notably, when exposed to αSYN-F, the astrocytes express all co-stimulatory markers CD40 (**e**), CD80 (**f**), and CD86 (**g**) that are crucial for activating CD4^+^ T cells. U, unstained samples; MFI, mean fluorescence intensity

Based on our findings that approximately half of the MHC-II-expressing cells in the PD brain are astrocytes, we next investigated whether astrocytes can act as antigen-presenting cells upon αSYN-F exposure. Subsequently, human astrocytes expressing the astrocytic markers GFAP, GLAST, ALDH1L1, vimentin, and S100β (Additional file 4) were exposed to αSYN-F for 7 days (Fig. 3a). Flow cytometry analysis revealed cell surface expression of MHC-II, MHC-I, and PDL-1 (Fig. 3b-d) upon stimulation with IFNγ. Interestingly, cell surface expression of the co-stimulatory molecules CD80, CD86, and CD40 (Fig. 3e–g) were only detected following exposure to sonicated αSYN-F. These results demonstrate that αSYN-F stimulate the astrocytes to express the co-stimulatory molecules necessary for professional APCs to activate CD4^+^ T cells.

### Intracellular deposits of αSYN in human astrocytes are surrounded by MHC-II

**Fig. 4:**
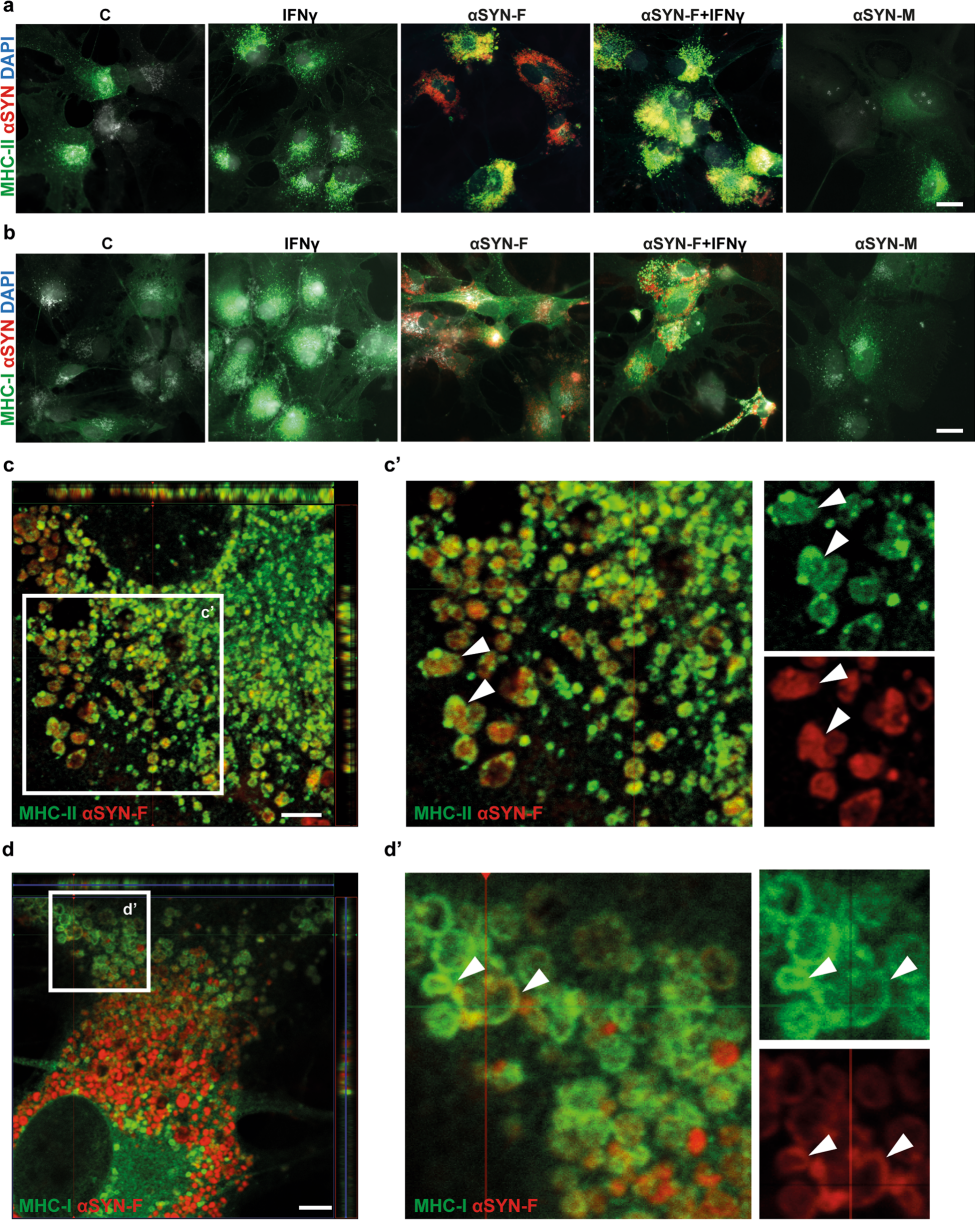
Intracellular deposits of αSYN in astrocytes are surrounded by MHC-II. Human astrocytes were exposed to αSYN-F for 7 days and stained for MHC-II and MHC-I. In the presence of IFNγ, the astrocytes clearly upregulate MHC-II (**a**) and MHC-I (**b**). The MHC-II totally enclosed the αSYN inclusions, which was confirmed with confocal microscopy (**c**). Close-up of white rectangle in **c** is shown in c’. Immunostaining for MHC-I analyzed with confocal microscopy showed a partial encampsulation of the αSYN deposits by MHC-I (**d**). Close-up of white rectangle in **d** is shown in d’. Scale bars: **a** and **b** = 20 μm, **c** and **d** = 5 μm

Professional APCs have the ability to present antigens with both MHC-II and MHC-I molecules, enabling them to activate both CD4^+^ and CD8^+^ T cells. In order to investigate whether αSYN-F and MHC-I/II are in close proximity in the human astrocytes, we performed immunocytochemistry. In line with our recently published data [15], the human astrocytes accumulated large amounts of aggregated αSYN, while monomeric αSYN was rapidly degraded by the cells (Fig. 4a, b). In accordance with the flow cytometry data, the number of cells expressing MHC-II and MHC-I in control cultures and αSYN-F-exposed cultures was similar. Stimulation with IFNγ increased the number of MHC-II positive cells as well as the expression of MHC-I (Fig. 4a, b) and PDL-1 (Additional file 5). Interestingly, in the αSYN and αSYN + IFNγ-treated astrocyte cultures, the majority of αSYN aggregates were surrounded by MHC-II . The localization of MHC-II, forming a capsule around the deposited αSYN, was confirmed with confocal microscopy (Fig. 4c, c'). These data together with the flow cytometry results strongly suggest an involvement of the MHC-II pathway in astrocytes when exposed to αSYN-F. Furthermore, immunostainings revealed MHC-I expression in the proximity of the intracellular αSYN deposits and partial encapsulation of αSYN was also observed for MHC-I, using confocal microscopy (Fig. 4d, d'). Since both MHC-I and II co-localize with αSYN aggregates, astrocytes might process αSYN as an antigen through both pathways, which further proposes their role as antigen presenting-cells.

### Intracellular αSYN and MHC-II are situated in LAMP-1 positive vesicles

**Fig. 5:**
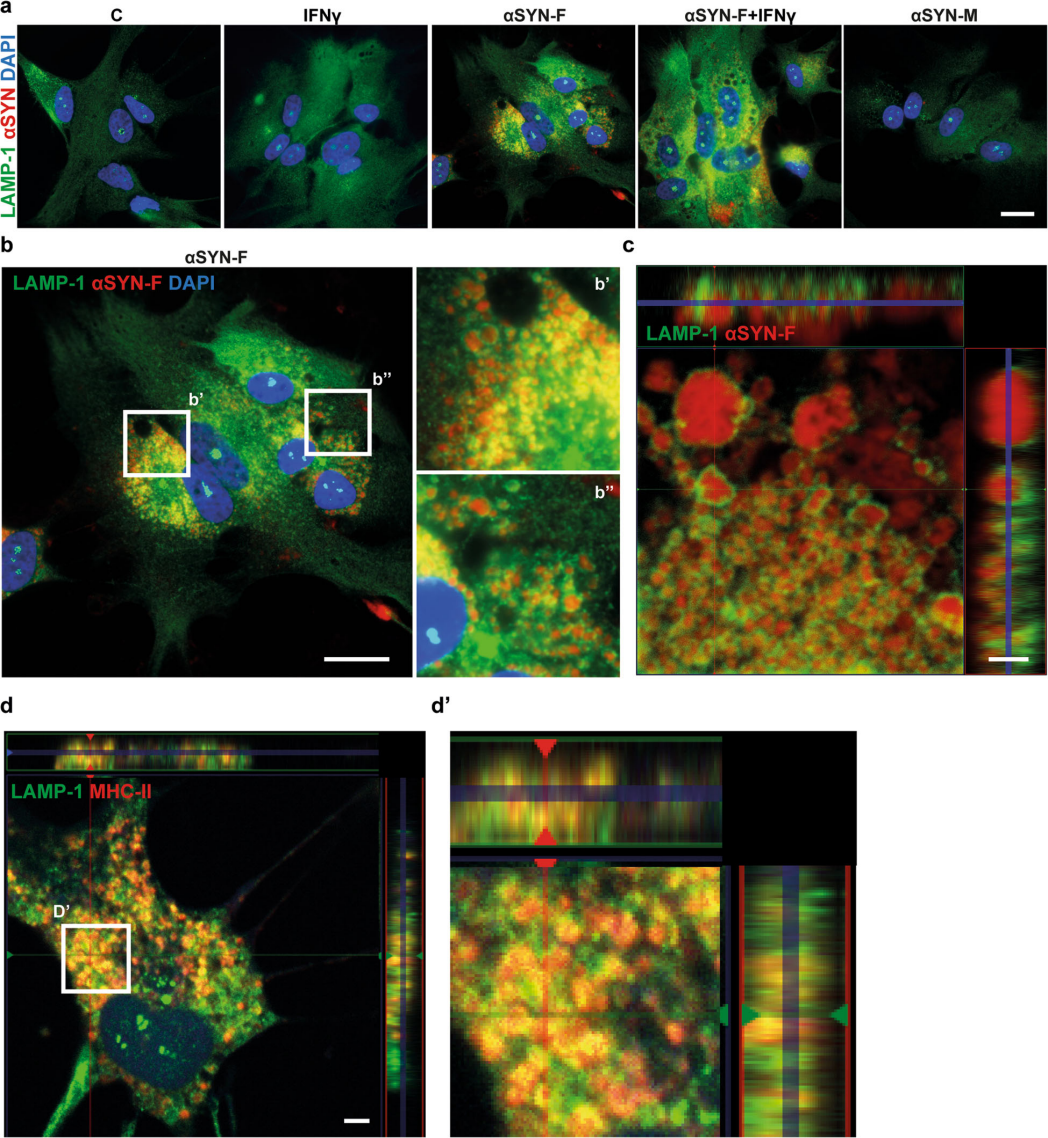
MHC-II and αSYN are located in LAMP-1 positive vesicles. Immunostainings showed that the LAMP-1 expression pattern was altered in αSYN-F exposed astrocytes, but not in astrocytes exposed to αSYN-M or IFNγ only (**a**). The αSYN deposits in αSYN-F exposed astrocytes are clearly localized within LAMP-1 positive vesicles (**b**). Close-up of white rectangles in b are shown in b’ and b”. Confocal analysis confirmed the localization of αSYN within the lysosomal compartments (**c**). Stainings for MHC-II and LAMP-1 in astrocytes, exposed to unlabeled αSYN-F, confirmed that also MHC-II is located in the LAMP-1 positive vesicles (**d**).Close-up of the white rectangle in **d** is shown in d’. Scale bars: **a**, **b** = 20 μm; **c** = 2 μm, **d** = 5 μm

MHC-II molecules encounter their antigen in late endosomal, lysosomal vesicles and are then transported to the cell surface. To find out if the αSYN deposits that are surrounded by MHC-II are within the same cellular compartment, immunocytochemistry with the lysosomal marker LAMP-1 was performed. The immunostainings revealed that the ingested αSYN-F enter the endo-lysosomal pathway (Fig. 5a, b). The localization of the αSYN deposits within LAMP-1 positive vesicles was confirmed by confocal microscopy (Fig. 5c). To find out whether the intracellular MHC-II is also surrounded by LAMP-1 positive vesicles, human astrocytes treated with unlabeled αSYN-F were stained with MHC-II and LAMP-1. Confocal microscopy demonstrated that LAMP-1 positive vesicles indeed surrounded the MCH-II molecules in the αSYN-F-treated astrocytes (Fig. 5d, d').

### Deposits of αSYN and MHC-II are transferred between astrocytes via tunneling nanotubes

**Fig. 6:**
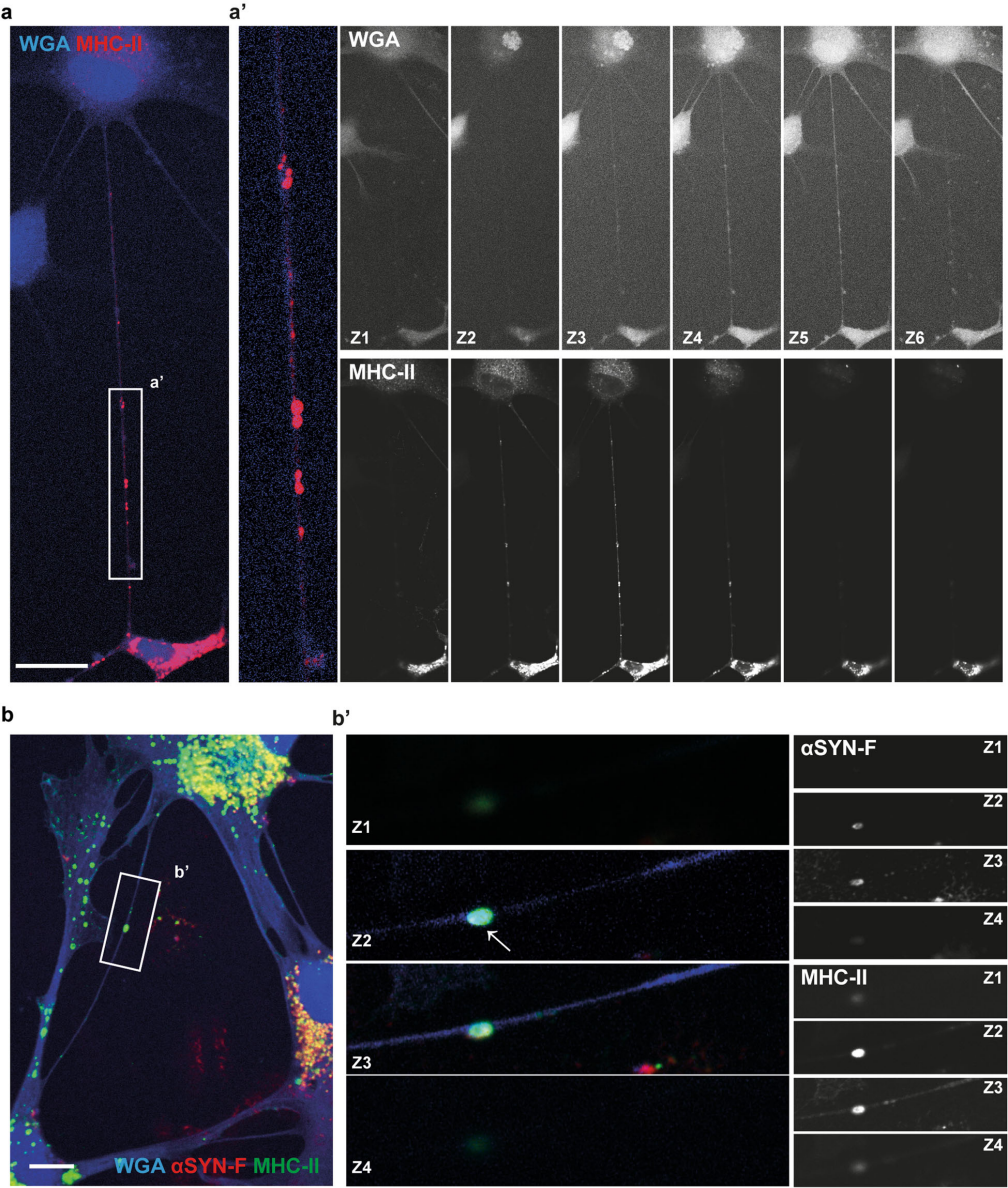
Aggregated αSYN and MHC-II are transported between astrocytes via tunneling nanotubes. Immunostainings and confocal imaging demonstrated transfer of MHC-II between the astrocytes via WGA-labeled tunneling nanotubes (**a**). A close-up of the tunneling nanotube region (white rectangle) is shown in a’. The various z-planes (z1–z6) for the WGA-staining and MHC-II-staining are shown separately in black and white. Aggregates of αSYN and MHC-II could be detected in tunneling nanotubes connecting two astrocytes (**b**). A close-up of the tunneling nanotube region (white rectangle) is shown in b’. The various z-planes (z1–z4) for the αSYN and MHC-II-staining are shown separately in black and white. Scale bars: (**a**) = 20 μm and (**b**) = 10 μm

Several studies have suggested that aggregated αSYN are transferred via tunneling nanotubes (TNTs) between astrocytes and between astrocyte and neurons [15, 25]. We wanted to explore whether MHC-II molecules also can spread between the human astrocytes and, if so, whether MHC-II and αSYN could be transported together from one cell to another. Immunostainings and confocal imaging showed that the MHC-II molecules were indeed transferred between the human astrocytes (Fig. 6a), suggesting that inflammatory molecules could be propagated from one astrocyte to another. Cell-to-cell transportation was detected in both control and αSYN-F exposed cultures. Additionally, confocal microscopy showed that the αSYN deposits surrounded by MHC-II also spread between the cells (Fig. 6b). These data suggest that the inflammation, as a part of the pathology, can spread from one astrocyte to another.

### MHC-II expressing astrocytes are situated in close contact with CD4^+^ T cells

**Fig. 7:**
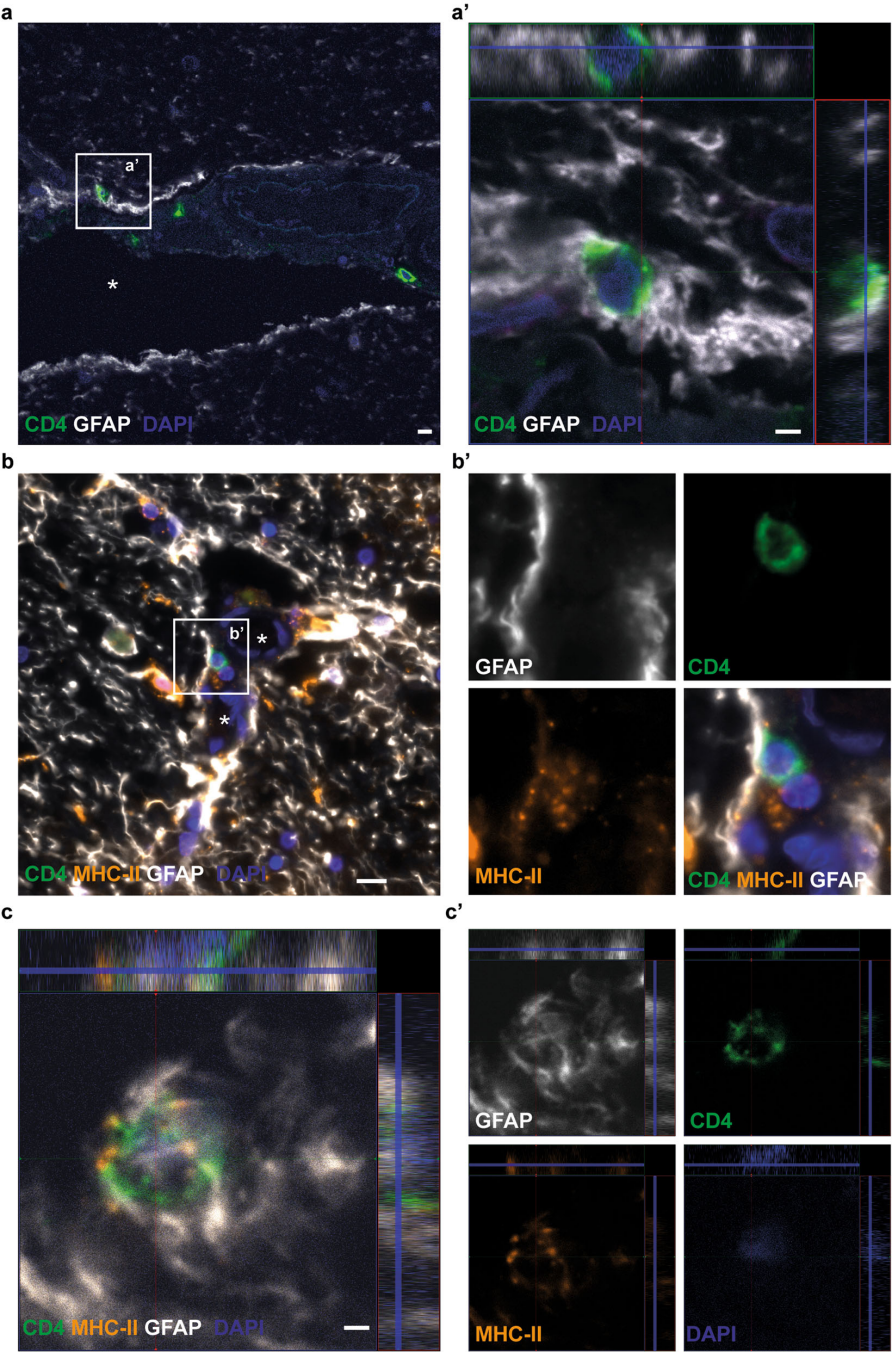
MHC-II expressing astrocytes are situated in close contact with CD4^+^ T cells in the human brain. Double immunostainings of GFAP and CD4 indicated close contact between perivascular CD4^+^ T cells and GFAP^+^ astrocytes in the vessel wall (**a**). A close-up confocal image of the white rectangle is shown in a’. Triple immunostainings with specific antibodies to GFAP, CD4, and MHC-II verified that there is an interaction between CD4^+^ T cells and MHC-II expressing astrocytes in the blood vessel wall (**b**). Close-up images of the separate channels are shown in b’. Infiltrated CD4^+^ T cells in the parenchyma were also found to be encapsulated by MHC-II expressing astrocytes (**c**). The separate channels of the confocal image are shown to the right in c'. The blood vessels in (**a**) and (**b**) are indicated with white stars. Scale bars: (**a** and **c**) = 2 μm, (**b**) = 20 μm

To examine whether astrocytes and CD4^+^ T cells are in direct contact with each other, human mesencephalon sections from PD post mortem brains were stained with specific antibodies to MHC-II, GFAP, and CD4. Infiltration of CD4^+^ T cells was observed in all the post mortem brain sections. Both perivascular and infiltrated T cells were demonstrated to be in contact with and closely surrounded by MHC-II expressing astrocytes (Fig. 7 a–c). Taken together, our data demonstrate that the astrocytes are capable of expressing all factors that are required for CD4^+^ T-cell activation, suggesting their involvement in T-cell activation during PD progression.

## Discussion

Accumulating evidence indicates that astrocytes can play a central role during PD progression. Reactive astrocytes are known to ingest large amounts of aggregated αSYN that are intracellularly stored, rather than degraded [8–14, 26]. We have previously shown that accumulation of aggregated αSYN in human astrocytes disrupts their lysosomal machinery and induces cell-to-cell transfer of αSYN via tunneling nanotubes [15]. Slow degradation of engulfed antigens is an important feature of professional APCs, enabling them to present antigens to T helper cells. Studies in multiple sclerosis and experimental autoimmune encephalomyelitis suggest that astrocytes are capable of activating both Th1 and Th17 T cells [27, 28], highlighting their central role in the inflammation process. However, no study has yet investigated the interplay between astrocytes and T cells in PD.

Here, we report for the first time that αSYN-accumulating astrocytes have the capacity to perform as professional APCs, indicating that they are important for the T cell response in PD. When exposed to fibrillary αSYN, human astrocytes express the co-stimulatory molecules CD80, CD86, and CD40 that are crucial for activation of CD4^+^ T helper cells, as well as MHC class I and II. Hence, the αSYN-accumulating astrocytes have all the molecules necessary for activating the infiltrating T cells. To confirm that astrocytes are in contact with CD4^+^ T cells in the PD brain, we performed immunostainings of post mortem tissues from PD patients. We detected CD4^+^ T cells within, as well as outside of the perivasculature. Interestingly, both the T cells situated in the vessel wall and in the brain parenchyma were found to be in very close proximity with MHC-II^+^ astrocytes. Since astrocytic end feet are lining the blood vessels, astrocytes could activate the T cells before or during their recruitment to the brain parenchyma.

Due to their myeloid origin, microglia are often suggested to be the primary APC in the brain, irrespectively of disease condition. However, several studies indicate that microglia are often poor antigen presenters [19–21], indicating that other glial cell types may instead be important for T-cell activation. For example, a recent investigation suggests that MHC-II-expressing oligodendrocyte progenitor cells (OPCs) may play a role in T-cell activation in multiple sclerosis [29]. Notably, we show that human microglia, isolated from adult brain tissue, do not express the co-stimulatory molecules necessary for T-cell activation upon αSYN-F exposure. Hence, our data indicate that astrocytes could be highly responsible for T-cell activation in the PD brain. Whether the astrocyte/T cell interplay is beneficial or detrimental for the disease progression, however, remains to be investigated.

Different types of T cells can be defined by the combinations of cell surface markers they express and the cytokines they secrete. Cytotoxic CD8^+^ T cells secrete IFNγ and are stimulated via the MHC-I pathway. The CD4^+^ T helper cells are divided into four subgroups: Th1, Th2, Th17, and regulatory T cells. These are activated through the MHC-II pathway and Th1/17 secrete IFNγ upon stimulation, whereas Th2 secrete IL-4 [30]. Alteration in the levels of Th1 and Th17 has been observed in the blood of PD patients [31–33]. Secretion of IFNγ by T cells stimulates expression of MHC-I, MHC-II, and the co-inhibitory molecule PDL-1 which is important for inhibition of T cells. To confirm that cultured human astrocytes express MHC-II and PDL-1 (that are regulated by IFNγ levels), the astrocytes were subjected to IFNγ. Immunostainings and flow cytometry analysis confirmed an increase of both endogenous and surface MHC-I, MHC-II, and PDL-1 in the astrocyte culture in response to IFNγ. Our findings indicate that astrocytes may be involved in the regulation of T cell function in the brain as they express both stimulatory and inhibitory molecules.

Antigens possess independent binding sites for both MHC-I and II molecules/complexes [34]. In addition to aggregation, αSYN undergoes different posttranslational modifications during disease progression, including phosphorylation [35, 36]. In a recent study, αSYN was suggested to have two MHC-II binding sites, one of which contains the ser129 phosphorylation site [3]. Immunostainings of post mortem mesencephalon tissue demonstrated higher levels of MHC-II in PD patients, compared to controls. Interestingly, the burden of ser129-phosphorylated αSYN in the mesencephalon of PD patients correlated with the MHC-II levels. Furthermore, we observed a correlation in MHC-II levels in striatum and mesencephalon in PD, but not in the controls. The nigrostriatal pathway, which includes mesencephalon and striatum, is heavily affected by αSYN pathology, explaining the correlation of MHC-II levels between the two regions. Consistently, another study found that the total burden of αSYN correlate with MHC-II in PD brain [37]. Immunostaining for GLAST, GFAP, and Iba1 indicated co-localization of both astrocytes and microglia with MHC-II in the PD brain. Subsequently, quantifications of the MHC-II + GLAST^+^ area revealed that 31% of total MHC-II, expressed in the mesencephalon sections, co-localized with the astrocytic marker, demonstrating that a high proportion of the MHC-II^+^ cells are indeed astrocytes.

In addition, 15% of the MHC-II area co-localized with GFAP and 32% co-localized with the microglia marker Iba1. The remaining MHC-II are most likely expressed by other cell types, such as macrophages situated in the blood vessels or by astrocytes that lack both GLAST and GFAP. Astrocytes are highly heterogeneous cells with different protein expression and different functions [38]. Interestingly, our staining shows that there is very little overlap between the GLAST and GFAP, indicating that the two markers identify different parts of the astrocytes and/or different astrocytic subclasses. Hence, in combination, our results show that almost 50% of the MHC-II is situated within astrocytes. It is also important to remember that GFAP only stains 15–20% of the astrocyte [24], which most likely results in a considerable underestimation of the MHC-II expressing GFAP^+^ astrocytes. Due to the limited number of patients included in the study, additional investigation of human brain material is necessary to fully understand the role of astrocytes in antigen presentation and T-cell activation in the PD brain. However, we performed extensive investigations of cultured human cells, confirming the antigen presenting capacity of astrocytes.

Endogenous antigens are presented via the MHC-I pathway where the antigen is fragmented to 8–10 amino acids in the proteasomal degradation pathway. Binding between MHC-I and the antigen occurs in the ER, and the antigen-MHC-I complex is then transported to the cell surface to interact with CD8^+^ T cells. Exogenous antigens that are ingested through endocytosis enter the endo-lysosomal pathway and are fragmented to 12–24 amino acids. These fragments are then presented to MHC-II, which is transported from the Golgi apparatus to the late endosomes where they bind to each other. The antigen-MHC-II complex is then transported to the cell surface, where it is presented to the CD4^+^ T helper cells [17]. Here, we show that the ingested αSYN fibrils are surrounded by MHC-II in LAMP-1 positive vesicles, suggesting that αSYN encounters MHC-II in the endo-lysosomes in astrocytes. Additionally, partial co-localization with MHC-I and αSYN could be observed, suggesting that the ingested αSYN-F could be released into the cytosolic compartment and enter the proteasomal pathway. Co-localization of αSYN aggregates by both MHC-I and II indicates that the astrocytes are capable of cross-presenting αSYN as an antigen. By sending parts of the antigen to the MHC-I pathway, professional APCs can present via both MHC-I and II molecules resulting in activation of both CD8^+^ and CD4^+^ T cells. These data indicate that αSYN fibrils, once ingested by the astrocytes, act as an antigen for the MHC-II pathway, but to some degree also for the MHC-I pathway.

We have previously shown that αSYN accumulation induce the formation of tunneling nanotubes between astrocytes [15]. The stressed astrocytes use the nanotubes to transfer αSYN to neighboring cells. Professional APCs have been suggested to transfer MHC-II molecules between each other via tunneling nanotubes [18]. We investigated whether MHC-II could be found in the tunneling nanotubes between the human astrocytes. Interestingly, not only MHC-II, but also αSYN/MHC-II complexes were transferred from one astrocyte to another via the tunneling nanotubes. Hence, astrocytes can possibly transfer αSYN deposits as an antigen bound to MHC-II to other astrocytes in the brain and thereby stimulate more astrocytes to become professional APCs.

## Conclusions

**Fig. 8:**
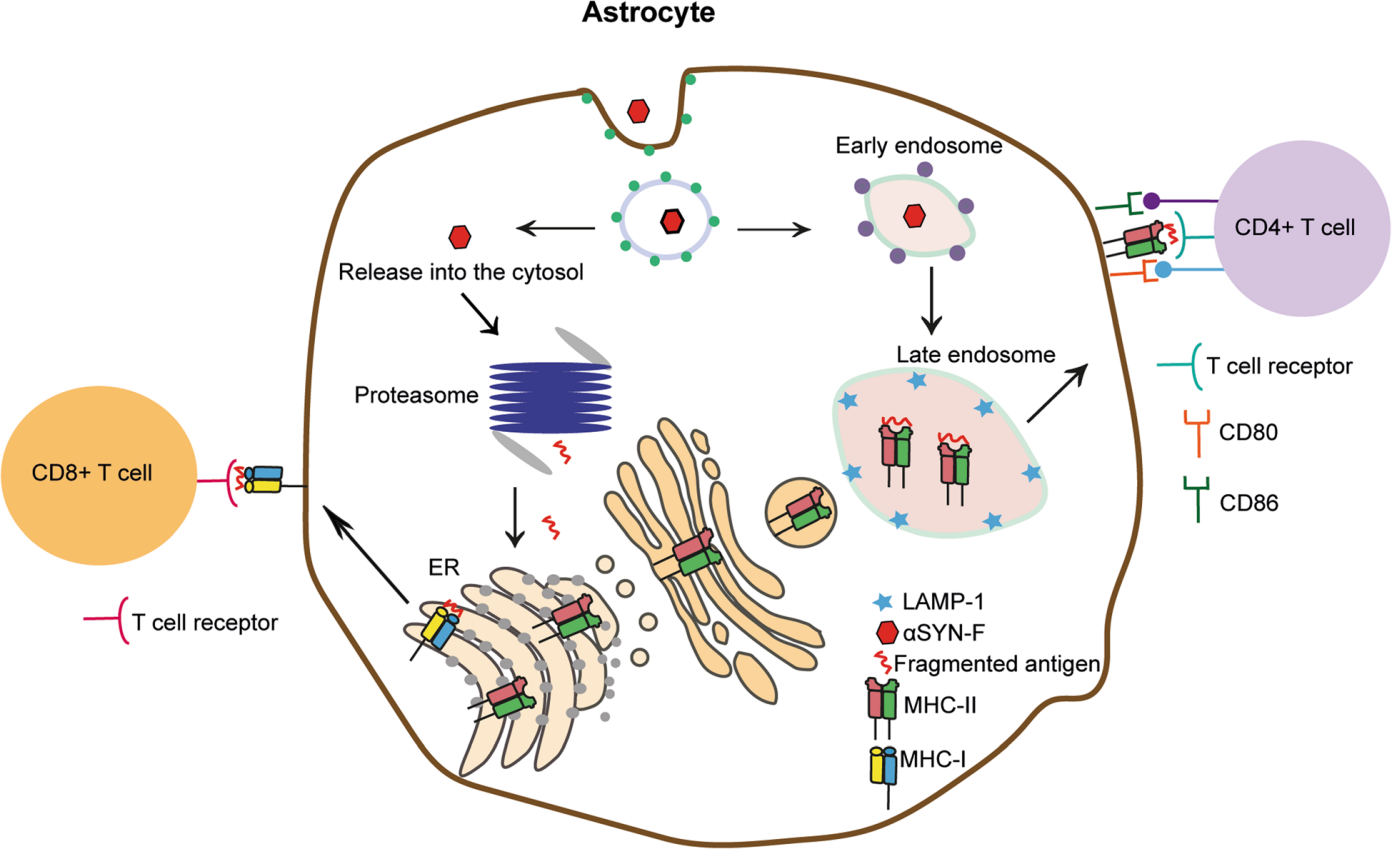
Proposed model for antigen presentation by astrocytes in the PD brain. Astrocytes ingest large amounts of αSYN-F, which can either enter the endo-lysosomal pathway or be released into the cytosol and processed via the proteasome pathway. Proteosomal digestion of the αSYN-F results in 8–10 aa long fragments that are transported to the ER to encounter MHC-I molecules. The MHC-I/αSYN complexes are then transported to the cell surface to be presented to CD8^+^ T cells. The αSYN-F that follow endo-lysosomal pathway are fragmented to pieces of 12–24 long aa that meet MHC-II in the late endosomes. Subsequently, The MHC-II/αSYN complexes are transported to the cell surface to be presented to CD4^+^ T cells. Activation of the CD4^+^ T cells requires surface expression of CD80 and CD86. Our results demonstrate that these receptors are only present on the human astrocytes that had engulfed αSYN-F.

Being the most numerous glial cell type in the central nervous system, astrocytes have great impact on brain homeostasis. Yet, their exact role in PD pathology remains elusive. Our histology and cell culture studies suggest that astrocytes have the potential to serve as major antigen-presenting cells in the PD brain. Following exposure to fibrillary αSYN, cultured human astrocytes, but not microglia, express the co-stimulatory molecules crucial for T-cell activation. Moreover, immunostaining of post mortem tissue confirms that MHC-II expressing astrocytes surround perivascular and parenchymal CD4^+^ T cells in the PD brain. Finally, aggregates of αSYN and MHC-II are transferred between astrocytes via tunneling nanotubes, suggesting a possible mechanism for spreading of αSYN-induced inflammation in the PD brain. In conclusion, our data indicate that astrocytes play an important role in antigen presentation and T-cell activation in the PD brain (Fig. 8), highlighting astrocytes as a promising therapeutic target in the context of chronic inflammation.

## Supplementary information

**Additional file 1.** MHC-II levels do not correlate with age in the analyzed material. Linear regression analysis on the MHC-II and the age in the PD group and in the control group demonstrate that there are no correlation between age and the expression of MHC-II in the material included in this study (61-84 years of age).

**Additional file 2.** Measurement of GLAST, GFAP and Iba1 in human PD and control brain sections. Integrated density of GLAST, GFAP and Iba1 was measured in mesencephalon sections of 10 PD and 10 control cases (a). Staining of GLAST and GFAP in mesencephalon revealed that these two astrocytic markers only overlap to some degree (b). Scale bar (b) = 20 μm.

**Additional file 3.** Human ES-derived astrocytes express astrocytic markers. Human astrocytes derived from embryonic stem cells express the astrocytic markers GFAP, ALDHL1, nestin, vimentin and GLAST. Scale bar = 20 μm.

**Additional file 4.** Characterization of pre-formed αSYN fibrils. ThT assay demonstrated β-sheet structure of the αSYN-F (a). TEM further confirmed the β-sheet structure of the αSYN fibrils and the reduction in fibril size after sonication (b-c). Scale bars: (b) = 2 μm, (c) = 200 nm.

**Additional file 5.** Astrocytes express PDL-1. When exposed to IFNγ, astrocytes express increased levels of PDL-1. Scale bar = 20 μm.

## Data Availability

All data generated or analyzed during this study are included in this article.

## Abbreviations

APC: Antigen-presenting cell
αSYN: Alpha-synuclein
CNTF: Ciliary neurotrophic factor
DC: Dendritic cell
ESC: Embryonic stem cell
GFAP: Glial fibrillary acidic protein
GLAST: Glutamate Aspartate transporter 1
Iba1: Ionized calcium-binding adaptor protein 1
IntDen: Integrated density
MHC: Major histocompatibility class
PD: Parkinson’s disease
PFA: Paraformal dehyde
ThT: Thioflavin T
TSA: Tyramide signal amplification
