# Supplementary figures and images for: Astrocytes have the capacity to act as antigen-presenting cells in the Parkinson’s disease brain
Source: J Neuroinflammation. 2020 Apr 16;17:119. doi: 10.1186/s12974-020-01776-7 (PMC7164247; doi:10.1186/s12974-020-01776-7)

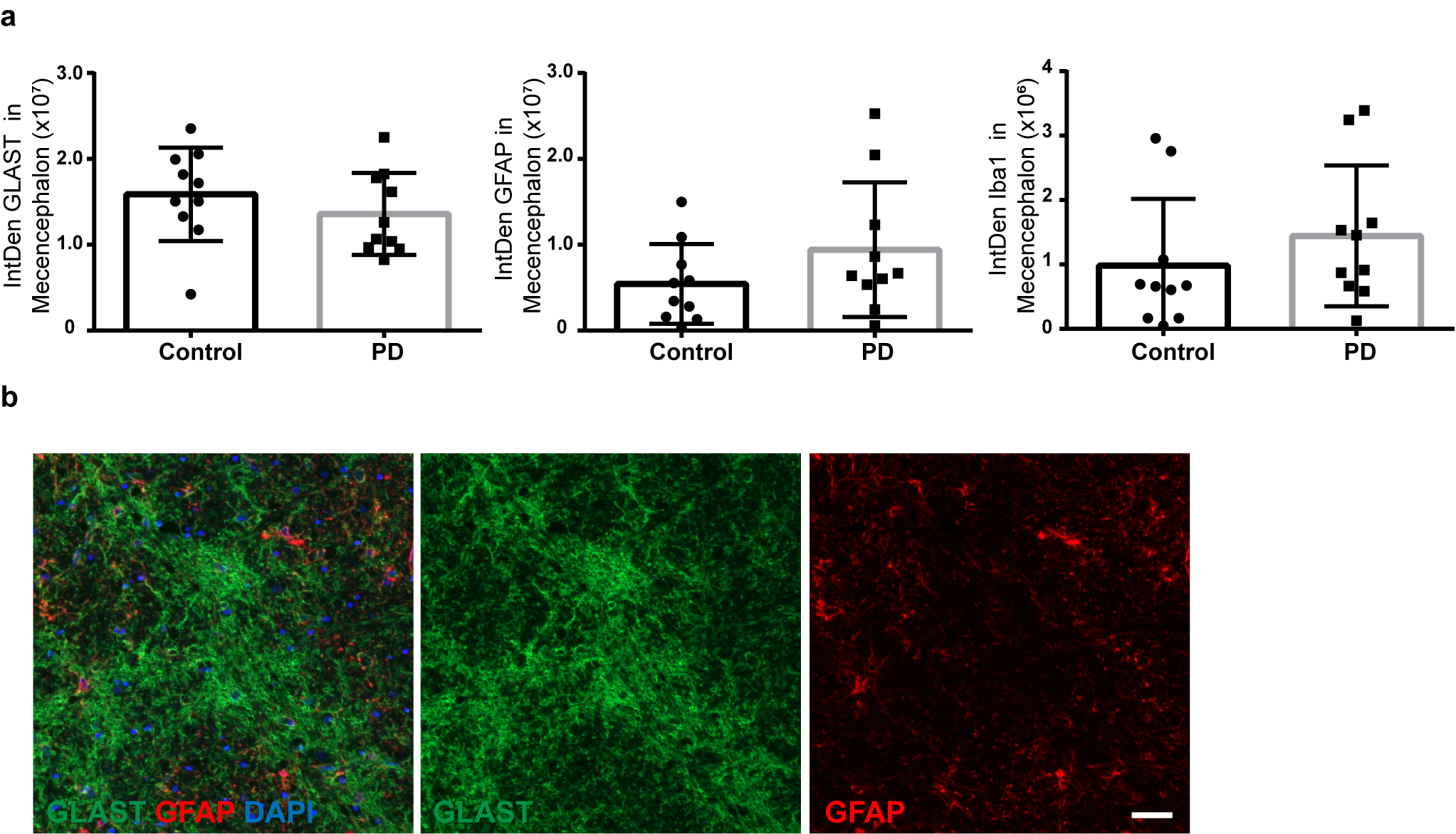

Supplement: Supplementary file 2 — Additional file 2. Measurement of GLAST, GFAP and Iba1 in human PD and control brain sections. Integrated density of GLAST, GFAP and Iba1 was measured in mesencephalon sections of 10 PD and 10 control cases (a). Staining of GLAST and GFAP in mesencephalon revealed that these two astrocytic markers only overlap to some degree (b). Scale bar (b) = 20 μm. [file 12974_2020_1776_MOESM2_ESM.tif]

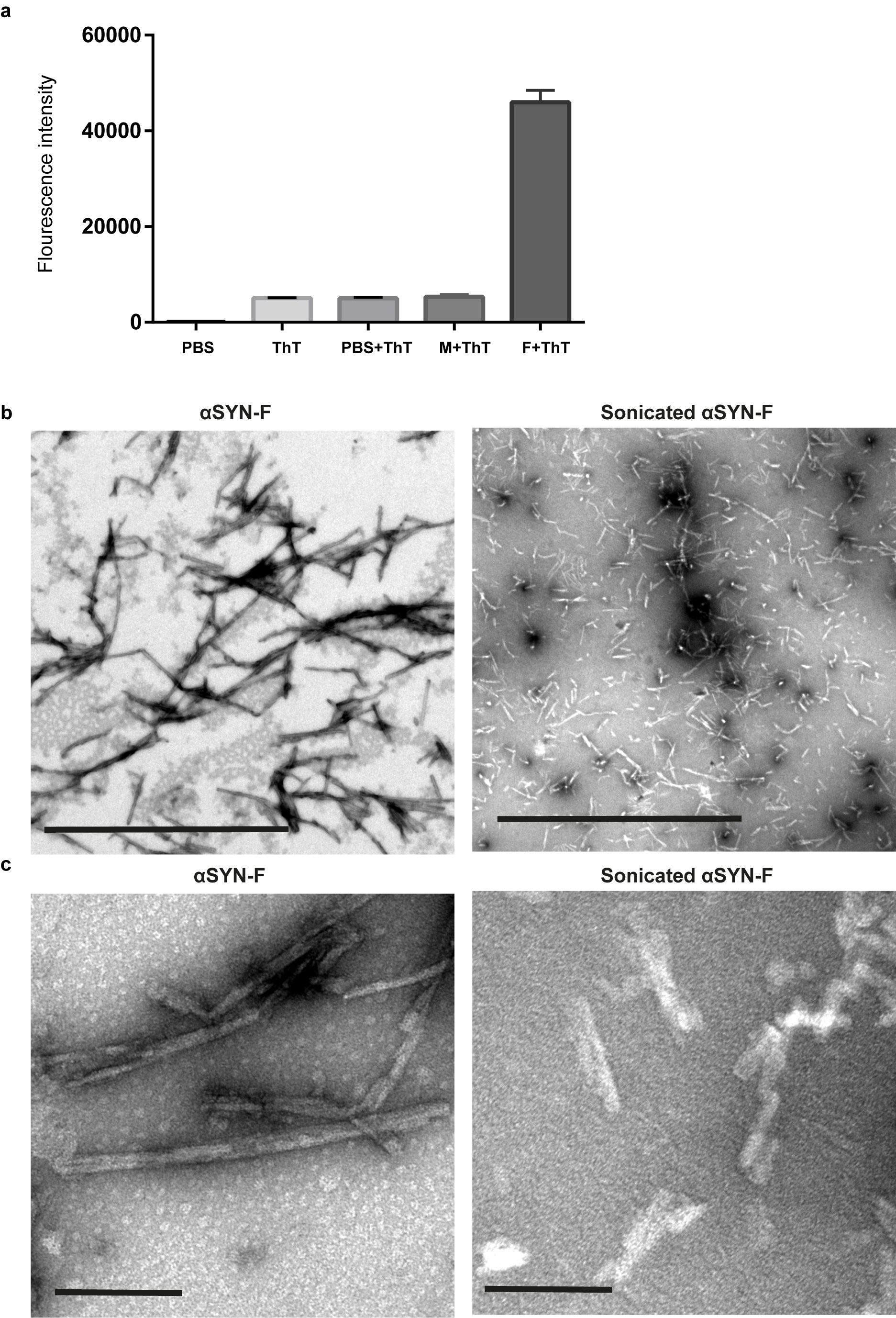

Supplement: Supplementary file 3 — Additional file 3. Human ES-derived astrocytes express astrocytic markers. Human astrocytes derived from embryonic stem cells express the astrocytic markers GFAP, ALDHL1, nestin, vimentin and GLAST. Scale bar = 20 μm. [file 12974_2020_1776_MOESM3_ESM.tif]
